# Cytoplasmic and Genomic Effects on Non-Meiosis-Driven Genetic Changes in *Brassica* Hybrids and Allotetraploids from Pairwise Crosses of Three Cultivated Diploids

**DOI:** 10.1371/journal.pone.0065078

**Published:** 2013-05-31

**Authors:** Cheng Cui, Xianhong Ge, Yingying Zhou, Maoteng Li, Zaiyun Li

**Affiliations:** 1 National Key Laboratory of Crop Genetic Improvement, National Center of Crop Molecular Breeding Technology, National Center of Oil Crop Improvement (Wuhan), College of Plant Science and Technology, Huazhong Agricultural University, Wuhan, China; 2 School of Life Science and Technology, Huazhong University of Science and Technology, Wuhan, China; CNRS UMR7275, France

## Abstract

Nuclear-cytoplasmic interactions are predicted to be important in shaping the genetic changes in early stage of allopolyploidization. Our previous study shows the specific role of genome and cytoplasm affecting the chromosome pairing in *Brassica* hybrids and allotetraploids from pairwise crosses between three cultivated diploids with A, B and C genomes, respectively. Herein, to address how parental genomes and cytoplasm affects genomic, epigenetic and gene expression changes prior to meiosis in these hybrids and allopolyploids, their patterns of AFLP (Amplified fragment length polymorphism), mAFLP (Methylation AFLP) and cDNA-AFLP were compared with the progenitors, revealing the major absent bands within each genome. These changes varied under various cytoplasm backgrounds and genome combinations, following the significant order of AFLP> mAFLP> cDNA -AFLP. The frequencies of AFLP bands lost were positively correlated with the divergence degrees of parental genomes, but not obvious for those of mAFLP and cDNA-AFLP absent bands, and methylation change showed least variations among hybrids and within each genome. These changes within each genome followed the A>B>C hierarchy, except the highest rate of cDNA loss in B genome. Among three changes, only overall AFLP bands were significantly correlated with cDNA-AFLP, and their correlations varied within each genome. These changes in allotetraploids were mainly caused by genome merger rather than doubling. Parental genomes altered differently at three levels, responded to the types of cytoplasm and genome and their interaction or divergence. The result provides new clues for instant non-meiosis-driven genome restructuring following genome merger and duplication.

## Introduction

Interspecific hybridizations and formation of alloployploids with genomes from two or more related parental species have contributed to the origins of many higher plants. The genetic consequences of genome merger have been investigated by utilizing the approaches of molecular biology and genomics in different systems of natural and synthetic allopolyploids, mainly including *Arabidopsis* [1]–[4], *Brassica* [5]–[7], cotton [8]–[9], *Triticale* [10]–[12], wheat [13]–[15] and other taxa *Tragopogon* [16], *Spartina* [17] and *Senecio* [18]–[19]. The dynamic nature of polyploid genomes is recognized [20], though the degrees of variations are markedly different between allopolyploids [21]–[22]. Widespread changes to gene expression in hybrids and allopolyploids are revealed by transcriptomic analysis, and genomic expression dominance occurs obviously in some systems [3], [23], [24]. It is considered that genomic plasticity has downstream effects on the transcriptome, proteome, and metabolome that can generate phenotypic variation in polyploids exceeding that found in the parents [20] or hybrid vigor [25], and may enable new hybrids to survive in novel habitats not accessible to their parent species [19].

The complex of six cultivated *Brassica* species in U-triangle is a model system for investigations of polyploidy. In reciprocal synthetics of *B. napus* and *B. juncea*, using the diploid species as maternal and paternal genome donors (AA.CC - CC.AA and AA.BB - BB.AA), the frequency of genetic change was influenced by the parental origin of the genomes, and the same genome showed more change when provided by the male parent [26]. Higher frequencies of genome change in *B. juncea* than in *B. napus* are considered to be associated with higher degrees of divergence between the parental A and B genomes than between A and C genome, or the nuclear-cytoplasmic incompatibility, because of the more closely related A and C cytoplasmic genomes and to the more compatible nuclear-cytoplasmic genomes in the AC and CA polyploids. In contrast, genetic changes in the S_5_ progenies of ∼50 resynthesized *B. napus* lines (CC.AA) occurred more frequently in the C-genome, while cytosine methylation changes occurred more frequently in the A-genome [6]; DNA fragment losses in this synthetics were significantly correlated with cDNA-AFLP fragment losses, but methylation changes were not correlated, suggesting that genomic rearrangements were largely responsible for loss of cDNA-AFLP markers. Consistently, the degree of C-genome-specific gene silencing and DNA methylation alterations are significantly greater than those of A-genome -specific alterations in another synthetic *B. napus* (AA.CC) [27]. Furthermore, a high proportion (25–38%) of polypeptides displayed quantitative nonadditive expression patterns, >60% of proteins being expressed in a manner similar to the paternal parent *B. rapa* [28]. But most of these proteins with a nonadditive pattern had additive transcript levels, suggesting that differential protein regulation is mainly governed by posttranscriptional modifications [29]. The alternative splicing changes of large scale are detected in natural and resynthesized *B. napus*, and are more common than homeolog silencing [30]. In other aspect, the cytoplasm background of resynthesized *B*. *napus* from its progenitors preferentially affects the transmission frequency of the meiotic-driven genetic changes to the progenies, and the *B. rapa* cytoplasm shows obvious suppressive role of meiotic rearrangements on the chromosomes of A genome [31]. The cytoplasmic and genetic effects on the meiotic crossover are also revealed in allohaploids produced from a wide range of *B*. *napus* accessions [32].

The present results available from natural and synthetic *Brassica* allopolyploids give the information that the cytoplasmic and nucleic genomes and their relatedness significantly affect the genetic and phenotypic performance of the allopolyploids formed, and that partaking genomes show the bias of their behavior at different levels [6], [26], [27], [28], [31]. To comprehensively elucidate the cytoplasmic and genomic effects on the genetics of the synthetic hybrids and allopolyploids, *Brassica* hybrids and allotetraploids have been produced from reciprocal pairwise crosses between three cultivated *Brassica* diploids, and their chromosome pairing quantified. The meiotic studies show that the pattern of intragenomic and intergenomic pairings in specific combinations highlight the specific role of genome and cytoplasm affecting their chromosome pairing [33]. In present study, to address how the parental genomes and cytoplasm affects genomic, epigenetic and gene expression changes prior to meiosis in these hybrids and allopolyploids, their patterns of AFLP, mAFLP and cDNA-AFLP are compared with the progenitors, which reveals the substantial alterations with major absent bands specific for each genome. These three types of changes occur at different extents and vary, depending on the cytoplasm background, genome combinations, genomes and relationships, and their interactions.

## Materials and Methods

### Plant Materials

The hybrids and allotetraploids used in this study were produced previously from reciprocal pairwise crosses between three cultivated *Brassica* diploids *B*. *rapa* (AA genome, genotype 3H120), *B*. *nigra* (L.) Koch cv. Giebra (2n = 16, BB genome), and *B*. *oleracea* var. *alboglabra* L. (CC genome, Chijielan). From each pair cross, the digenomic diploid and triploid hybrids were produced, A.B/BB.A, A.C/CC.A, B.C/CC.B. Three allotetraploids (AA.BB, AA.CC and BB.CC) were synthesized by doubling the chromosome numbers of the respective digenomic hybrids (A.B, A.C and B.C), and CC.AA allotetraploid was directly obtained from the cultured embryo-plantlet, probably by the spontaneous chromosome doubling *in vitro*. Two trigenomic hybrids (A.C.B and C.A.B) were obtained from the crosses between the synthesized *B*. *napus* (AA.CC/CC.AA) and *B*. *nigra* [33].

### DNA, RNA Extraction, and cDNA Synthesis

To ensure the homogenous identity of each hybrid or allotetraploid, at least five plantlets from one embryo of each cross were produced by successive subculturing on the MS [34] medium with 1.5 mg/L 6-benzyl aminopurine (6-BA), 0.25 mg/L α-naphthalenacetic acid (NAA) and then transferred in the field. About two months later, the freshly expanded leaves of the cloned young plants from each hybrid and allotetraploid were collected and pooled for AFLP and mAFLP. For gene expression analysis, the cloned plantlets of these synthetics on the medium above were maintained in the growth chamber for 14 days under 14h of light and 10h of darkness at 24°C, and then the fresh leaves were collected for RNA extraction. Total DNA was isolated using the Cetyltrimethyl ammonium bromide procedure [35], and total RNA was extracted by using Trizol reagent (Invitrogen, Life Technologies) according to manufacturer’s protocol. Before reverse transcription, ∼20 µg RNA was treated with RNase-free DNase I (Fermentas, China) at 37°C for 30 min to avoid genomic DNA contamination. First-strand cDNA was synthesized with Oligo dT(18) by using RevertAid First strand cDNA synthesis Kit according to the manufacturer’s recommendations (Fermentas K1622, China). Second-strand cDNA was synthesized using 10 U DNA polymerase I and 3 U RNase H according to the manufacturer’s protocol (Fermentas, China). The produced double-stranded cDNA was purified by phenol-chloroform extraction and ethanol precipitation, and then resuspended in a final volume of 12 µl ddH_2_O, 2 µl was checked on an agarose gel. If the expected smear between 100 and 4000 bp was observed, the rest of the cDNA was stored at −20°C for further use.

### DNA-mAFLP Analysis

The Methylation AFLP analysis was performed following the general procedure [36] with minor modification, using the enzyme combination *Pst*I/*Mse*I. Cytosine methylation is predominately present at CpG or CpNpG sites [37]. *Pst*I is highly sensitive to the cytosine status in CpNpG sites because its recognition site (CTG CAG) involves two CpNpG trinucleotides. The adaptors and primers sequence specific for these enzymes are listed in Table S5. Genomic DNA (0.5 µg) was digested with 5 U *Pst*I and 5 U *Mse*I (New England Biolabs) with adding 50 ng/µl BSA (New England Biolabs) for 5 h at 37°C. The 10 µl of a solution was then added, containing 5 pmol of AFLP *Pst*I adapter*, 50 pmol *Mse*I adapter, 50 pmol *Mse*I-adapter^+^, 1 U T_4_ DNA-ligase (Fermentas, China), and the mixure was incubated for 12 h at 22°C. After ligation, the reaction mixture was diluted to 1∶5 with ddH_2_O and a ‘whole-genome amplification’ step was carried out with two non-selective *Mse*I primers corresponding to two *Mse*I adapters (see Table S5). Amplification mixture contained 5 µl template DNA, 30 ng *Mse*I primer, 30 ng *Mse*I primer+, 0.4 U *Taq* polymerase. PCR reactions were performed under 3 min at 94°C, 7 cycles consisting of 30 s at 94°C, 1 min at 56°C, 2 min at 72°C, and a final extension step of 10 min at 72°C. After amplification, the reaction mixture was digested again with 5 U *Pst*I in 40 µl for 5 h at 37°C. Next, 5 pmol *Pst*I adapter and 1 U T_4_ DNA ligase was added and the mixture was incubated at 22°C for 12 h. After ligation, the reaction mixture was diluted to 1∶30 with ddH_2_O and then the standard AFLP pre-selective and selective amplifications were performed [38].

### DNA and cDNA-AFLP Analyses

The AFLP analysis was performed according to Vos [38] and the cDNA-AFLP technique was modified from published methods [39]–[40]. Simply, ∼100 ng of DNA or cDNA was digested with 5 U *Mse*I and 5 U *Eco*RI (New England Biolabs) for 6 h at 37°C. The digestion was inactivated by heating for 15 min at 70°C and then the digested fragments were ligated to *Eco*RI and *Mse*I adaptors by T_4_ DNA ligase (New England Biolabs). The pre-amplification step was carried out using 5 µl of diluted ligation products (1∶10) as templates, with 0.2 µM *Eco*RI and *Mse*I pre-selection primers, in a final volume of 25 µl containing 1× polymerase chain reaction (PCR) buffer, 1.5 mM MgCl_2_, 0.2 mM dNTP and 2 U *Taq* polymerase (Fermentas). The PCR amplification was performed with the following cycle profile for 24 cycles: a 30 s DNA denaturation step at 94°C, a 60 s annealing step at 56°C and a 60 s extension step at 72°C. The pre-amplification product was diluted 1∶15 with ddH_2_O, and 2 µl was used as template for selective amplification. The components of reaction mixture were the same as above but with pair of selective primers. Selective PCR amplification was performed with the touchdown PCR profile: 94°C for 3 min, 12 cycles of 30 s denaturing at 94°C, 30 s annealing at 65°C (reduced 0.7°C/cycles) and 60 s extensions at 72°C, followed by 25 cycles of 30 s denaturing at 94°C, 30 s annealing 56°C and 60 s extensions at 72°C, ending with 10 min at 72°C to complete extension. As negative controls for DNA contamination in the RNA samples, reactions were also performed without RT (RNA samples treated with RNase-free DNase I, starting from digestion-ligation step), side-by-side with the experimental reactions.

### PCR Products Separation

Selective PCR products in DNA-, methylated DNA- and cDNA-AFLP were mixed with 10 µl of formamide dye, denatured at 100°C for 5 min and separated by electrophoresis on 6% denaturing polyacrylamide. The gels were pre-run at 100 W for about 30 min before 3 µl of the mix was loaded, then run at 80 W for ∼ 1 h. Silver staining was performed according to the normal procedure (Promega).

### Scoring and Statistical Analyses of AFLP, mAFLP and cDNA-AFLP Bands

Only clear and identifiable bands in AFLP, mAFLP and cDNA-AFLP gels were used for scoring. The scored bands were transformed into a binary character matrix, using “1″ and “0″ to indicate the presence and absence, respectively. To compare the difference of same marker changes in the same genome in two different hybrids and the difference of the same marker changes in different genomes in one hybrid, two-by-two chi-squared contingency tests were performed. Logistic regressions analysis using linear models were performed to compare the correlations among total AFLP, mAFLP and cDNA-AFLP marker changes simultaneously in different hybrids. All the analysis was performed by Microsoft Excel (2007).

## Results

### Genomic Changes in Hybrids and Allotetraploids

The genetic changes in hybrids and allopolyploids were detected by the comparison between parents and polyploid gel profiles [12]. From 59 pairs of AFLP selective primers, 614 to 830 bands were amplified in three progenitors with A, B and C genomes (Table S1). In A genome, 59 (9.61%) and 173 (28.18%) bands shared with those in B and C genomes, respectively. In B genome, 59 (7.51%) and 112 (14.25%) bands were common to those in A and C genomes, respectively. In C genome, 173 (20.84%) and 112 (13.49%) bands were common to those in A and B genomes, respectively. A, B and C genomes shared 84 bands, while conserved 298 (48.53%), 531 (67.56%) and 461 (55.54%) genome-specific bands, respectively. The bands shared between A and C genomes (173) were higher than those between B and C (112), and between A and B (59) genomes, which confirmed their closer relationship.

**Figure 1:**
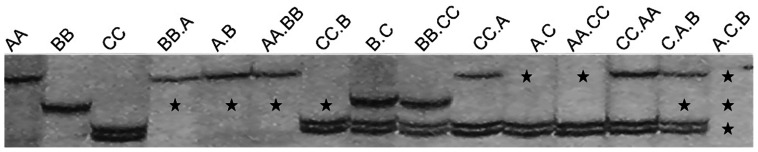
AFLP patterns of hybrids and allotetraploids compared with three progenitors AA, BB and CC. ★: absent parental bands.

**Figure 2:**
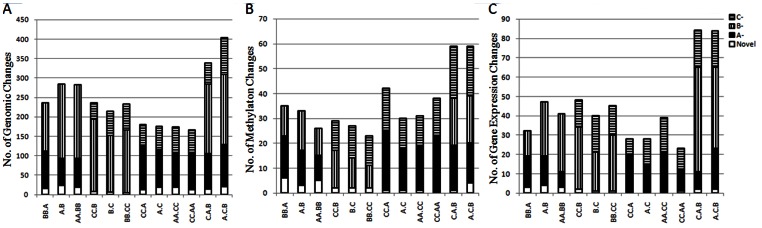
Number of genome-specific absent bands and novel bands for AFLP (A), mAFLP (B) and cDNA-AFLP (C) in hybrids and allotetraploids. Novel: Novel bands; A^−^, B^−^, C^−^: genome-specific bands lost in A, B and C genomes, respectively.

From these primers, 1199 to 1738 bands were amplified in the hybrids and allotetraploids, which included the absent bands specific for each genome or shared by two or three genomes, and also the novel bands, besides the conserved parental bands (Fig. 1). The total number and percentage of their absent bands were much higher than those of novel bands (Fig. 2a). The percentage of averaged absent bands in A and B genomes was similar but higher than that in C genome (Table 1 and Table S2). For the ratio of the total bands (absent bands and novel bands)(17.10%–29.14%), B.C, BB.CC, A.C, AA.CC, CC.A, CC.AA and C.A.B had similar rates, being significantly lower than those in A.C.B and BB.A, and then lower than those in A.B, AA.BB. So BB.A, A.B and AA.BB with two most distantly related A and B genomes had similar but highest rates, those with A and C or B and C genomes had the lowest rates, the triploid A.C.B had the intermediate rate (Table 1).

**Table 1:** Percentages of genome-specific absent bands and novel bands for AFLP, mAFLP and cDNA-AFLP in hybrids and allotetraploids.

Combinations	AFLP					mAFLP					cDNA-AFLP				
	Total	A^−^	B^−^	C^−^	Novel	Total	A^−^	B^−^	C^−^	Novel	Total	A^−^	B^−^	C^−^	Novel
BB.A	25.79*^bc^*	32.55*^bc^*	23.35*^a^*	–	1.56	24.40*^c^*	30.91^ab^*	16.22^a^*	–	3.66	7.62*^ab^*	13.68^ab^*	6.84^a^*	–	0.51
A.B	29.14*^c^*	23.49*^a^*	35.97*^c^*	–	2.47	19.42*^abc^*	25.45^ab^*	21.62^a^*	–	1.78	9.98*^ab^*	12.82^ab^*	14.74^bc^*	–	0.70
AA.BB	28.76*^c^*	24.83*^a^*	35.78*^c^*	–	2.05	18.27*^abc^*	18.18^a^*	14.86^a^*	–	2.86	7.94*^ab^*	6.84*^a^*	15.79*^bc^*	–	0.51
CC.B	20.66*^a^*	–	35.03*^c^*	9.11*^a^*	0.70	15.56*^abc^*	–	20.27^a^*	11.21^a^*	0.91	11.27*^ab^*	–	16.84*^bc^*	7.78*^ab^*	0.32
B.C	17.95*^a^*	–	27.50*^ab^*	13.45*^b^*	0.51	11.67*^ab^*	–	16.22^a^*	12.15^a^*	0.87	8.39*^ab^*	–	10.53^ab^*	10.56^b^*	0.16
BB.CC	19.37*^a^*	–	30.32*^bc^*	14.53*^b^*	0.43	10.12*^a^*	–	12.16^a^*	11.21^a^*	0.86	8.54*^ab^*	–	15.26*^bc^*	8.33*^ab^*	0.16
CC.A	19.02*^a^*	38.26*^c^*	–	11.71*^ab^*	1.22	21.94*^c^*	43.64*^b^*	–	15.89*^a^*	0.54	4.86*^a^*	17.09*^b^*	–	4.44*^a^*	0
A.C	17.33*^a^*	32.55*^bc^*	–	12.80*^ab^*	1.87	15.61*^abc^*	30.91*^ab^*	–	11.21*^a^*	0.50	7.78*^ab^*	12.82^ab^*	–	7.22^ab^*	0
AA.CC	18.01*^a^*	30.20*^ab^*	–	14.32*^b^*	1.79	17.72*^abc^*	32.73*^ab^*	–	11.21*^a^*	0.51	8.27*^ab^*	17.95^b^*	–	10.00^ab^*	0
CC.AA	17.10*^a^*	32.55*^bc^*	–	12.36*^ab^*	1.19	19.49*^bc^*	41.82*^b^*	–	14.02*^a^*	0	9.39*^ab^*	9.40^ab^*	–	6.11^ab^*	0.18
C.A.B	20.74*^a^*	30.87^abc^*	33.71^c^*	11.71*^ab^*	0.94	19.94*^c^*	32.73^ab^*	25.68^a^*	19.63^a^*	0.40	13.10*^b^*	7.69^a^*	28.42*^d^*	10.56^b^*	0.28
A.C.B	24.57*^b^*	36.24^bc^*	34.09^c^*	20.61*^c^*	01.5	19.43*^bc^*	29.09^ab^*	25.68^a^*	18.69^a^*	1.56	12.73*^b^*	17.95^b^*_’_ **	22.11^cd^**	10.56^b^*	0.28
Average	21.61	31.28*	31.97*	13.40	1.31	17.70	31.72	19.09	13.91	1.13	9.34	12.92	16.32	8.40	0.26

A^−^, B^−^, C^−^: specific fragments lost belonging to *Brassica* A, B, C genomes, respectively; *^a,b,c,d,^*: Groups in each column significantly different by *χ^2^* -test, *P*<0.05; The * indicates that the percentages of the change from different genomes in each hybrid in each row are insignificantly different (the chi-square test, α = 0.05), respectively. The ** revealed by cDNA-AFLP in A.C.B were insignificantly different (the chi-square test, α = 0.05).

In three digenomic diploids (A.B, B.C, A.C) and two trigenomic triploids (A.C.B, C.A.B) which had genomes in haploidy state, the number and percentage of the absent bands in B genome were significantly higher than those of A or C genomes in A.B and B.C, and the values in A genome were higher than C genome in A.C; but in A.C.B and C.A.B, the absent bands in C genome were lower than those in B and A genomes, the latter two had no obvious difference. In three digenomic triploid hybrids (BB.A, CC.A and CC.B) which contained one genome in diploidy state and another in haploidy state, the absent bands in the haploidy genome were higher than those in the diploidy genome, especially in CC.A and CC.B.

As A, B, C genomes in these hybrids were from the same three diploids, the genomic alterations within the same genome in different hybrids could be compared, which should reveal how the alterations varied, depending on the genome combinations and the cytoplasm type. The percentage of absent bands within haploidy A genome in A.B was significantly lower than those in A.C, BB.A, CC.A, A.C.B, but not lower than that in C.A.B, while the values of all other five had insignificant differences. The significant higher rate in A.C than A.B was unexpected, for both had the cytoplasm of *B. rapa*, and A and B genomes were more distantly related than A and C genomes. The lower rate in A.B than BB.A resulted possibly from the different types of cytoplasm or from the haploidy or diploidy B genome. The similar trend occurred in A.C and CC.A, for the rate of A genome in CC.A was insignificantly higher than in A.C. For the percentages of haploidy B genome, B.C with the cytoplasm of *B. nigra* presented a lower rate than those in A.B, CC.B, A.C.B and C.A.B, in which no significant difference was found each other. Once more, the value in B.C was lower than that in CC.B. For the comparison of the haploidy C-genome specific absent bands, the percentages in A.C, B.C and C.A.B had no significant differences but were significantly lower than that in A.C.B. In these hybrids, the number of novel bands was low and varied in narrow ranges (6–23). Similarly, the shared absent bands detected were much fewer than genome-specific absent bands, particularly those shared by three genomes in C.A.B and A.C.B. Two trigenomic triploids A.C.B and C.A.B showed similar percentage of absent bands in A and B genomes, but A.C.B gave higher value in C genome than C.A.B, suggesting that C genome changed more in the A-type cytoplasm of *B. rapa*. In the comparison between A.B and A.C.B/C.A.B, A genome in triploids lost more than in diploid, especially in A.C.B, but B genome exhibited similar rates of loss. So the loss of A genome was enhanced in triploids with the addition of C genome. Between A.C and A.C.B/C.A.B, A genome lost at similar rates, but C genome in A.C showed similar rate with C.A.B but significantly lower than A.C.B. So the more loss of C genome was also possibly caused by the addition of B genome, for A.C and A.C.B had the same cytoplasm. Between B.C and A.C.B/C.A.B, B genome in diploid lost at significantly lower rate than in two triploids, but C genome in diploid had the loss at similar rate with that in C.A.B but significantly lower than in A.C.B.

In three pairs of digenomic diploids and triploids (A.B/BB.A, B.C/CC.B, A.C/CC.A) produced from reciprocal pair crosses of three diploids (AA, BB and CC), the same haploidy genome in triploids showed significantly more absent bands than in diploids; inversely, another genome in diploidy state in triploids had significantly fewer absent bands than that in haploidy state in diploids, except for C genome in A.C/CC.A. The differences of percentages of total changes between A.B and BB.A or between B.C and CC.B were larger than that between A.C and CC.A. The number of novel bands in diploids was higher than that in triploids in A.B/BB.A and A.C/CC.A, but was nearly the same in B.C/CC.B. These data suggested that the genome in diploidy state triggered more genomic loss in another genome in haploidy state, and the diploidy state of one genome was more stable than its haploidy state. The cytoplasm also probably contributed to the differences of genomic changes, because the paternal genome in maternal cytoplasm exhibited higher percentage of absent bands, especially in more distantly related genomes (A and B, B and C). The higher number of novel bands in diploids than in triploids also hinted that the genomes in haploidy state altered more.

In synthesized allotetraploids, B genome showed much higher number and percentage of absent bands than A or C genome in AA.BB and BB.CC. In the reciprocal synthetics AA.CC/CC.AA, A genome gave much higher values of absent bands than C genome, but the value in each genome had no significant difference between the two allotetraploids, though A or C genomes in alien cytoplasm had a little higher rate, showing the minor effect of the two closely related cytoplasm types in enhancing the change of paternal genome in this combination. A genome showed a lower rate of absent bands in AA.BB than in AA.CC/CC.AA, which was in contrast with other study [26]. B genome in AA.BB had an insignificant higher rate than in BB.CC, but C genome had the similar rates in AA.CC/CC.AA and BB.CC. Between BB.A and AA.BB, B genome in diploidy state in the BB.A showed a rate of absent bands significantly lower than in AA.BB, and the same for C genome between CC.B and BB.CC, but lower insignificantly for C genome in CC.A than in AA.CC. Such differences showed that the genomes in diploidy states also lost more bands in alien cytoplasm and the loss was likely reduced by the haploidy state of another genome. Inversely, A genome in haploidy state had a significantly higher rate than that in diploidy state between BB.A and AA.BB, between CC.A and AA.CC, but insignificantly higher for B genome in CC.B than in BB.CC. These differences also showed that the genomes lost more in alien cytoplasm and the loss was likely reduced by the diploidy state of another genome, or the balanced dosage of parental genomes could reduce the genomic changes.

The comparison of genomic changes between hybrids and resultant allotetraploids should reveal the effect of genome duplication. Between A.B and AA.BB, B.C and BB.CC, A.C and AA.CC, each pair showed no significant difference in the percentage of absent bands within two genomes, and only exhibited limited increase of absent and novel bands in allotetraploids. These allotetraploids also lost some bands of the respective hybrids and gained some new bands. AA.BB had nearly the same rates of loss and gain, but AA.CC and BB.CC had higher rates of loss than gain, while their total rates of both loss and gain were similar (Table 2). This result suggested that the genome merger was mainly responsible for the genomic change and genome duplication had secondary effects on genomic change.

**Table 2:** Percentages of bands loss or gain for AFLP, mAFLP and cDNA-AFLP in synthetic allotetraploids compared with respective hybrids.

Allotetraploids	AFLP			mAFLP			cDNA-AFLP		
	Loss %	Gain %	Total %	Loss %	Gain %	Total %	Loss %	Gain %	Total %
AA.BB	2.24	2.16	4.31	7.25	7.73	13.94	1.31	3.05	4.29
AA.CC	2.44	1.53	3.90	4.76	2.65	7.17	4.73	4.24	8.59
BB.CC	2.34	0.93	3.23	2.85	4.4	7.00	2.22	2.37	4.49

Loss: bands absent in allotetraploids compared with respective hybrids; Gain: bands novel in allotetraploids compared with respective hybrids; % gain = novel/(novel+ no change)×100; % loss = (absent bands)/(absent bands+additive bands)×100.

For the comparison of absent bands within A genome in nine hybrids and allotetraploids related, those with the cytoplasm of *B. rapa* (A.B, AA.BB, AA.CC) had the lowest but similar rates, A.C and A.C.B had the rates with no significant difference with AA.CC. CC.A had the highest rate but was similar with BB.A, CC.AA and C.A.B, though these rates were also similar with A.C and A.C.B, suggesting that the A genome in alien cytoplasm showed more loss. The lower values in A.B and AA.BB than in A.C and AA.CC, which all had cytoplasm of *B. rapa*, were the opposite reported by Song [26], for the more distant relationship between A and B genomes than between A and C genomes were considered to cause more genetic change in synthetic *B. juncea* than *B. napus*. For the values within B genome in eight hybrids and allotetraploids, BB.A had the lowest percentage but was insignificantly different from B.C, while the other six (A.B, AA.BB, BB.CC, CC.B, A.C.B and C.A.B) had highest but similar rates. So B genome changed less in the cytoplasm of *B. nigra* rather than in that of *B. rapa* and *B. oleracea*. The rate in BB.A (23.35%) was much lower than that in A.B (35.97%), showing the impressive effect of the cytoplasm and genome dosage on stabilizing or disturbing genomes. For the C genome in nine hybrids and allotetraploids, CC.B had the lowest percentage but was insignificantly different from those in CC.A, A.C, CC.AA and C.A.B, while A.C.B had the highest rate, B.C, BB.CC and AA.CC had the rates higher than CC.B. Again, C genome was more stable in the cytoplasm of *B. oleracea*, but responded less in alien cytoplasm than A and B genomes. The significantly higher rate in B.C than in CC.B also suggested the effects of the cytoplasm and genome dosage on genomic alteration.

The absent bands shared by two or three genomes occurred at lower rates than the genome-specific absent bands. The rates of absent bands shared between any two genomes were much higher than that of three genomes, and the rate of AB genomes was higher than that of AC or BC genomes.

### DNA Methylation Changes

From 21 pairs of mAFLP selective primers, 111 to 172 bands were amplified in three progenitors. In A genome, 9 (8.11%) and 30 (27.03%) bands shared with those in B and C genomes, respectively. Among 118 bands in B genome, 9 (7.63%) and 18 (15.25%) were common to those from A and C genomes, respectively. In 172 bands of C genome, 30 (17.44%) and 18 (10.47%) were common to those in A and B genomes, respectively. A, B and C genomes shared 17 bands, while had 55 (49.55%), 74 (62.71%) and 107 (62.21%) genome -specific bands, respectively (Table S1).

From those mAFLP selective primers, a total of 206 to 330 bands were amplified in these hybrids and allotetraploids, involving the absent and novel bands with the former being much more frequent (Fig. 2b). In average, A genome had the higher rate (31.72%) than B (19.09%) and C genomes (13.91%) (Table 1 and Table S3). For the total methylation changes, the percentages ranged from 10.12% in BB.CC to 24.40% in BB.A. BB.CC had the lowest rate, being significantly lower than those in CC.AA and A.C.B, and then lower than those in BB.A, CC.A and C.A.B. The highest rates in BB.A and CC.A originated mainly from different dosages of two parental genomes, less from the genome relatedness. It seemed that those with B and C genomes had lower rates than those with A and B or A and C genomes, and the correlation between the rate of mAFLP loss and genome relatedness was not obvious.

The rates of the genome-specific absent bands in each hybrid showed the hierarchy A>B>C, but the differences between any two genomes were significant only in CC.A, A.C, AA.CC and CC.AA with more loss for A genome. For the rates within each genome among hybrids, A genome showed significant differences, but not for both B and C genomes, which suggested that cytoplasmic backgrounds and genome combinations had profound effects on the methylation change of only A genome. Furthermore, only the lowest rate within A genome in AA.BB was significantly lower than those in CC.A and CC.AA, while the rest of hybrids and allotetraploids with A genome showed unobvious differences, indicating that the methylation of A genome also responded not very sensitive on the cytoplasm and genomes, and that the rates were negatively correlated with divergence of parental genomes, the reverse of the genomic change. However, A genome tended to show lower rates in those with the cytoplasm of *B. rapa* (A.B, AA.BB, A.C, AA.CC, A.C.B) (Table 1).

In hybrids with all genomes in haploidy state (A.B, B.C, A.C, A.C.B, C.A.B), the absent bands of the same genome in each hybrids had no significant difference. In BB.A, CC.A, CC.B, the absent bands in the haploidy genome were generally higher than those in the diploidy genome, but the difference was significant only in CC.A. The rates of absent bands within haploidy A genome gave no significant differences in the hybrids involved (BB.A, A.B, CC.A, A.C, A.C.B, C.A.B). The lower rate of A genome-specific absent bands in A.B than BB.A, and in A.C than CC.A, suggested that alien B or C -type cytoplasm or dosage of B or C genome had some effect on the loss of A genome. Though B genome exhibited no obvious differences of the absent bands in different hybrids, it still produced more in alien cytoplasm, particularly in triploids A.C.B and C.A.B. The haploidy C-genome also lost more in triploids A.C.B and C.A.B. The shared absent bands occurred much fewer than genome-specific absent bands (Table 1 and Table S3).

In three pairs of digenomic diploids and triploids (A.B/BB.A, B.C/CC.B, A.C/CC.A), the same haploidy genome in triploids still showed more but insignificant absent bands than those in diploids, another genome in diploidy state in triploids had insignificant fewer absent bands than in haploidy state in diploids, except for C genome in A.C/CC.A (Table 1 and Table S3). In synthesized allotetraploids, A genome gave significantly higher rates of absent bands than C genome in the reciprocal synthetics AA.CC/CC.AA, but two parental genomes lost at similar levels in AA.BB and BB.CC. However, A or C genome gave the similar rates between AA.CC and CC.AA, though A genome had some higher rate in CC.AA than AA.CC. Of interest, A genome in AA.BB lost at much lower rate (18.18%) than in CC.AA (41.82%), but was insignificantly lower than in AA.CC (32.73%). B genome in AA.BB had little higher rate than in BB.CC, but C genome had the similar rates in AA.CC/CC.AA and BB.CC. Between BB.A and AA.BB, CC.B and BB.CC, and CC.A and AA.CC, the genome in diploidy state in triploids and allotetraploids had similar rates; inversely, the genome in haploidy state in triploids had a higher but insignificantly rate than its diploidy state in allotetraploids.

For the methylation changes between diploid hybrids and resultant allotetraploids, each pair showed no significant difference in absent bands within each genome, but the diploid hybrids had little higher rates than the allotetraploids in A.B/AA.BB and B.C/BB.CC, not in A.C/AA.CC. In these allotetraploids, loss of some bands of the respective hybrids and gain of some new bands occurred, more frequently in AA.BB. AA.BB had nearly the same rates of loss and gain, but AA.CC had higher rate of loss than gain and BB.CC showed the reverse (Table 2).

The percentages of absent bands shared by two or three genomes were comparable, but much lower than that of A-genome-specific absent bands, and some lower than those of B and C genomes (Table S3).

### Gene Expression Changes

From 37 pairs of cDNA-AFLP selective primers, 375 to 472 bands were amplified in three progenitors. In A genome, 28 (7.47%) and 105 (28.00%) bands shared with those in B and C genomes, respectively. Among 405 bands in B genome, 28 (6.91%) and 62 (15.31%) were common to A and C genomes, respectively. In 472 bands of C genome, 105 (22.25%) and 62 (13.14%) were common to A and B genomes, respectively. A, B and C genomes had 117 (31.20%), 190 (46.91%) and 180 (38.14%) genome-specific bands, respectively, while they shared 125 bands (Table S1).

From these primers, 617 to 809 bands were amplified in hybrids and allotetraploids, which also included the absent bands specific for each genome or shared by two or three genomes, and also the novel bands, besides the conserved parental bands. Only 0–4 novel bands appeared, but the genome-specific absent bands had much higher numbers (8–54) (Fig. 2c). Totally, the average percentage of B genome (16.63%) was significantly higher than that of A (12.92%) and C (8.40%) genomes, and that of A genome was higher than C genome (B>A>C). For the total change of cDNA bands (4.86% to 13.10%), CC.A had the lowest rate, significantly lower than those in A.C.B and C.A.B, but not lower than all others, suggesting that the merger of three genomes caused more change of gene expression, mainly loss (Table 1 and Table S4).

Among diploids (A.B, A.C, B.C) and triploids (A.C.B, C.A.B) with all genomes in haploidy state, C.A.B had the lowest rate of the absent bands in A genome and also had significant difference with that of A.C.B, which was in contrast to the expectation of the cytoplasmic effect; A.B and A.C had the same rates within A genome without significant difference with those of A.C.B and C.A.B. The rate of B genome in B.C was lowest but not significantly different with that in A.B, while significantly lower than those in A.C.B and C.A.B. The rate in C.A.B was some higher than in A.C.B, but insignificantly. Notably, the rates of B genome in triploids were obviously higher than those in diploids. The rate of C genome in CC.A was lowest and significantly different from those in B.C, A.C.B and C.A.B, but not from those in CC.B, BB.CC, A.C, AA.CC and CC.AA; A.C.B and C.A.B had the same rates. In the comparison between A.B/A.C/B.C and A.C.B/C.A.B, A genome in the triploid A.C.B had higher but insignificantly rates than in A.B and A.C; B genome in triploids showed significant higher rates than in A.B and B.C, except between A.B and A.C.B; C genome in two triploids had the same rate as in B.C, little higher than in A.C. In BB.A, CC.A and CC.B, the absent bands in the haploidy genome were higher than those in the diploidy genome, and the difference was significant in CC.A. A genome changed little more in CC.A than in BB.A, and C genome lost more in CC.B than in CC.A. B genome in CC.B showed significantly more loss than in BB.A (Table 1 and Table S4).

To compare the percentages of absent bands within haploidy A genome, the frequency in C.A.B was lowest and significantly lower than those in CC.A and A.C.B, but not significantly lower than those in BB.A, A.B, A.C. The one with the cytoplasm of *B. rapa* showed lower but insignificantly rate for A genome in pairs of hybrids (A.B/BB.A, A.C/CC.A), but the reverse for A.C.B/C.A.B. For the percentages of haploidy B genome, A.B, CC.B and B.C had similar rates, being significantly lower than those in A.C.B and C.A.B. Within haploidy C genome, A.C, B.C, A.C.B and C.A.B had rates without significant differences, showing that the cytoplasmic background and genome combination had limited effects on gene expression. Two triploids A.C.B and C.A.B showed similar values in B and C genomes, but B genome changed more than in other hybrids, and also unexpectedly, A genome in A.C.B lost much more than in C.A.B.

In three pairs of digenomic diploids and triploids (A.B/BB.A, B.C/CC.B, A.C/CC.A), the same haploidy genome in triploids showed more but insignificant absent bands than those in diploids; the genome in diploidy state in triploids had fewer absent bands than in haploidy state in diploids, especially in A.B/BB.A. In synthesized allotetraploids, B genome showed higher number and percentage of absent bands than A or C genome in AA.BB and BB.CC, especially in AA.BB. In the reciprocal synthetics AA.CC/CC.AA, A genome gave insignificantly higher rate than C genome, but the rates in each genome between the two allotetraploids had no significant difference. AA.BB showed a significantly lower rate of A genome than AA.CC, not CC.AA, which was in contrast with other study [26]. B genome in AA.BB had very similar rate with BB.CC, and C genome had the similar rates in AA.CC/CC.AA and BB.CC. Between BB.A and AA.BB, B genome in diploidy state in BB.A showed a rate significantly lower than in AA.BB, but lower insignificantly for C genome between CC.B and BB.CC, and between CC.A and AA.CC.

In hybrids and resultant allotetraploids (A.B and AA.BB, B.C and BB.CC, A.C and AA.CC), each pair showed no significant difference in absent bands within two genomes. A genome in AA.BB had a lower rate (6.84%) than in A.B (12.82%), and C genome in BB.CC (8.33%) also had a lower rate than in B.C (10.56%). In these allotetraploids, loss of some bands of the respective hybrids and gain of some new bands were more frequent in AA.CC (Table 2). AA.CC and BB.CC had nearly the same rates of loss and gain, but AA.BB had higher rate of gain than loss (Table 2). This result suggested that the genome duplication induced further change of gene expression.

For the absent bands within A genome in nine hybrids and allotetraploids related, the correlation with the cytoplasm of *B*. *rapa* was inconsistent, because AA.BB and C.A.B showed the similar but lowest rates, while A.C.B, AA.CC and CC.A kept the similar but highest rates, which were significantly higher than those in AA.BB and C.A.B. In reciprocal synthetics with A and B genomes, A.B and BB.A had similar rates, which were nearly double of the rate in AA.BB, but still insignificantly. In reciprocal synthetics with A and C genomes, AA.CC and CC.A had very close rates and were two times higher than that of CC.AA. The lower values in AA.BB than in AA.CC contrasted those reported by Song [26]. Within B genome in eight hybrids and allotetraploids related, BB.A had the lowest percentage, being significantly lower than others (A.B, AA.BB, BB.CC, CC.B, A.C.B and C.A.B), but not than B.C. A.B, AA.BB, BB.CC and CC.B had similar rates, which were much lower than that in C.A.B. Within C genome in nine hybrids and allotetraploids, CC.A had the lowest rate of absent bands with significant differences with those in B.C, A.C.B and C.A.B, but not with those in A.C, AA.CC, CC.AA, BB.CC and CC.B. The results also showed the B and C genome responded inconsistently to the cytoplasm backgrounds and genome combinations.

The absent bands shared by two or three genomes occurred at lower rates than the genome-specific absent bands, the rates of any two genomes were comparable but higher than that of three genomes.

### Correlations among Losses of DNA, mDNA and cDNA Fragments

**Figure 3:**
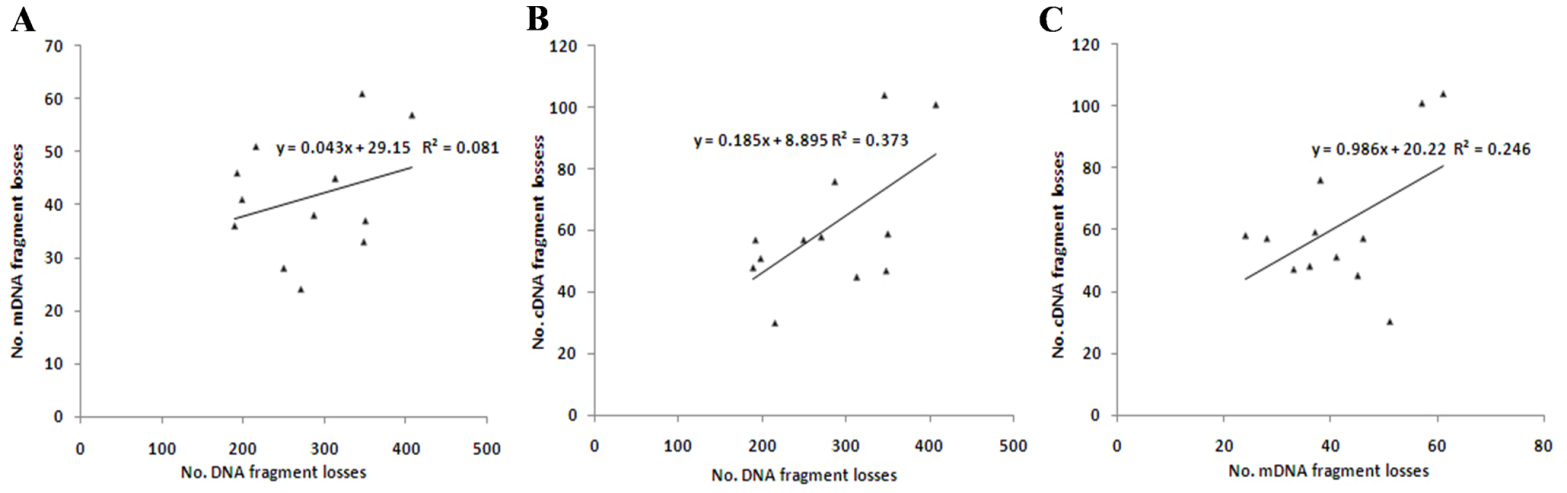
Relationships between total loss number of AFLP, mAFLP and cDNA-AFLP bands in hybrids and allotetraploids. Correlation between AFLP and mAFLP fragment losses (A), correlation between AFLP and cDNA-AFLP fragment losses (B), correlation between mAFLP and cDNA-AFLP fragment losses (C).

In these hybrids and allotetraploids, absent DNA bands were significantly correlated with absent cDNA bands (F = 7.13>F_0.05(1, 10)_), not with absent mDNA bands (F = 0.87<F_0.05(1, 10)_), and also absent mDNA bands were not significantly correlated with absent cDNA bands (F = 3.26<F_0.05(1, 10)_; Fig. 3). Within A genome, the correlation coefficient between absent DNA and mDNA bands was significant (F = 8.01>F_0.05(1,7)_), but not for those between DNA and cDNA bands (F = 3.55<F_0.05(1,7)_) or between mDNA and cDNA bands (F = 0.74<F_0.05(1,7)_). Within B genome, absent mDNA and cDAN bands (F = 6.41>F_0.05(1,6)_) was significantly related, but absent absent DNA and cDNA bands (F = 4.25<F_0.05(1,6)_) or DNA and mDNA bands (F = 1.51<F_0.05(1,6)_) were not. Within C genome, three types of absent bands were not significantly correlated, but the coefficient between mDNA and cDNA bands (F = 0.26<F_0.05(1,7)_) were lower than those between DNA and mDNA (F = 0.9<F_0.05(1,7)_), and between DNA and cDNA bands (F = 1.89<F_0.05(1,7)_). These results showed that the loss of DNA fragments was largely responsible for loss of cDNA fragments, but was differently related to mDNA loss in three genomes, and also that the loss of mDNA was variably correlated with the loss of cDNA.

The averaged percentage of absent and novel AFLP bands (21.61%) was higher than that of mAFLP (17.70%) and than cDNA-AFLP (9.34%) in these hybrids and allotetraploids, and such trends existed within three genomes, except that A genome had nearly the same rates of AFLP and mAFLP bands. A and B genomes had the very similar rates of AFLP absent bands but much higher than C genome, but A genome showed a much higher rate for mAFLP absent bands than B and C genomes, and also B genome had a significant higher rate than C genome. For the loss of cDNA, B genome had a significant higher rate than A and C genomes, and A genome had the higher rate than C genome. So A genome presented the highest rates of genomic and methylation changes, but not gene expression, B genome had the highest rate of genomic and expression losses but the second rate for methylation, while C genome gave the lowest rates of these changes which varied within narrower ranges than those of A and B genomes. This also indicated that the correlations among these changes were different in the three genomes.

## Discussion

Two major types of genetic changes have been revealed to be often associated with nascent plant allopolyploidy: loss, disappearance of parental bands and gain, appearance of novel bands. The detection of a loss event is dependent on the nature of the bands being polymorphic between the two parental species, but a gain event will be detectable irrespective of parental polymorphism or monomorphism. The loss and gain events indicate the occurrence of genome rearrangements, but some of them may be not “real” genetic changes associated with allopolyploidization, resulting from PCR competition between the divergent parental species DNA [41]. Whereas these sequence changes prior to meiosis in F_1_ hybrids and newly synthesized allotetraploids are widespread [1], [2], [5], [11]–[14], [21], the mechanisms behind remain largely unknown [19].

In this study, by using the same type of molecular marker on one set of hybrids/allopolyploids from three *Brassica* diploids, the general trends of the genetic changes prior to meiosis were compared, which gave interesting information for the effects of the cytoplasm and genome on these changes. The substantial alterations in genome component, methylation and gene expression prior to meiosis were revealed to appear with the decreasing extents in the serial synthesized *Brassica* hybrids and allotetraploids. These changes responded differently to the types of cytoplasm and genome and their interaction or divergence, with genomic change being most sensitive but the methylation most insensitive. Each parental genome also showed different degrees of these changes with the hierarchy A>B>C genomes, except the highest rate of cDNA loss in B genome. These three types of changes were differentially correlated for overall and within each genome. The changes in allotetraploids were attributable mainly to genome merger and secondarily to genome doubling. These results provides new clues for instant non-meiosis-driven genome restructuring following genome merger and duplication, as no mechanism for DNA elimination is currently accepted.

### Cytoplasmic and Genomic Effect on Genetic Changes in Hybrids and Allopolyploids

As shown by the first study demonstrating extensive and rapid genomic changes accompanying *Brassica* polyploid formation [26], the cytoplasm background and genome divergence or their interaction affected the genetic changes in our hybrids and allopolyploids. Those with A and B genomes showed significantly higher rates of total genomic changes including absent and novel bands than those with A and C or B and C genomes, while the latter had the similar rates (Table 1), as A and B genomes shared fewer AFLP bands than the other two genome pairs (Table S1). Interestingly, two trigenomic hybrids (A.C.B/C.A.B) presented higher rates than those with A and C or B and C genomes but lower than those with A and B genomes, indicating that the incorporation of B genome caused more changes but not to the level of those with most distant genomes. Moreover, the hybrid A.C.B had significantly higher rate than C.A.B, revealing that these genomes, particularly C genome changed more in the A-type than C-type cytoplasm. The correlations of the genome divergence and cytoplasm with the changes of DNA methylation and gene expressions were not as obvious as the genomic change. Though BB.A had the highest rate of mAFLP, but was not significantly higher than all with A and C genomes and two triploids (A.C.B, C.A.B) or CC.B, while those with B and C genomes showed lowest rates. For cDNA-AFLP, all those but two trigenomic triploids had comparable rates, and CC.A had the lowest rate. Two trigenomic triploids showed some higher rates but not significantly different from most of the others.

The total extents of three genetic changes were different under various cytoplasm backgrounds and genome combinations, following the patterns of genomic DNA>mDNA>cDNA. So the components of parental genomes were most sensitive to the change of cytoplasm and genome merger, probably because they were the direct target of genome shock from interspecific hybridization [42]; while the methylation status of the parental genomes was most insensitive, for B and C genomes showed similar loss rates in all hybrids and allotetraploids. For the changes within each genome, the hierarchy was A>B>C genome, except the equal rates of genomic loss between A and B genomes, and the higher rate of cDNA loss in B than A genome. Even more, the divergence degrees of the cytoplasm and genomes had the correlation with the rates of three types of changes in inverse directions: a positive correlation with genomic loss within each genomes, and a negative correlation with methylation change (obviously in A genome, for B and C genomes had similar rates), and also a negative correlation with cDNA loss within A genome mainly, and seemingly within B genome, but somehow no correlation within C genome. These results indicated that responses of parental genomes in these hybrids and polyploids to the cytoplasm types and genome combinations varied at the different levels of genetic changes, in different genomes, to the nuclear-cytoplasmic interaction.

The frequencies of genomic loss in our allotetraploids were roughly in parallel with those from Song et al. [26], except for the lower rate in C genome, possibly for they observed the differences of RFLP patterns between single F_2_ plants and their self-pollinated F_5_ progenies, while we studied the difference of AFLP patterns between F_1_ hybrids/derived allotetraploids and their parents. Their allopolyploids might accumulate more genetic changes by meiotic rearrangements during successive generations, such as homoeologous nonreciprocal transposition in resynthesized *B. napus* [6]. The comparative sequence analysis between homoeologous genome segments of natural *B*. *napus* and its two progenitor species also revealed that the C genome is more vulnerable to undergo changes than A genome after its formation [43]. But the genomic loss in our hybrids and allopolyploids occurred prior to meiosis, resulting from genome merger and doubling. This also showed that the decrease in DNA content in the present day *Brassica* alloploid species likely started on their synthesis [44]–[45].

The three pairs of the hybrids and resultant allotetraploids (A.B and AA.BB, B.C and BB.CC, A.C and AA.CC) showed the similar percentages of three types of genetic changes, revealing that the hybridization of divergent genomes other than the genome doubling made the main contributions to the genetic restructuring, as observed in amphihaploid and amphidiploid *B. napus* [18] and other systems [19], [46]. Some allotetraploids gave lower rates in some genomes than the hybrids, though insignificantly. Whether there was immediate calming (ameliorating) effect of polyploidization on changed levels of gene expression experienced by the hybrid in *Senecio* system [18] needs further study. Some obvious differences of the changes within some genomes between the three pairs (AA.BB and BB.A, BB.CC and CC.B, AA.CC/CC.AA and CC.A) hinted the effects of genome dosage or the genome imbalance on the genetic changes, together with the cytoplasm. Of course, compared with the hybrids, these allotetraploids also showed some different changes by losing some bands or gaining some novel bands. It was expected that the genomes in these allotetraploids would be further restructured through meiotic recombination and activation of transposable elements caused by the genome shock [6], [43].

### Relationships between Genetic and Epigenetic Changes and Gene Expression

The significant correlation between the loss of AFLP and cDNA-AFLP bands, but insignificant between the loss of AFLP and mAFLP bands or between mAFLP and cDNA-AFLP bands in these synthetic hybrids and allotetraploids meant that the lost DNA sequences involved the parts of the genes unexpressed, but not the large parts of methylation loci, and also the loss of methylation loci was not directly responsible for the change of gene expression. Furthermore, the variable relationships among three genetic changes within three genomes suggested that each genome followed different regulations to modify its genomes at three levels of DNA sequences, DNA methylation and transcription in response to genome merger and doubling. Such difference in genome behavior might be related to the differences in genome structure. The inconsistent levels of three changes in each hybrid and allotetraploid possibly were attributable to the effect of cytoplasm and genome combination, which seemed to be related to their divergence, as A.C and AA.CC with most closely related cytoplasmic and nucleic genomes showed consistent levels of total change rates (Table 1).

In several different synthetic allopolyploids, nonadditive gene expression, biased expression of homeologs, and homeologous gene silencing are shown to occur after polyploidization events [47]–[50], and these phenomena have been referred to as transcriptome shock [19], [51]. Rapid and widespread changes to the overall methylation profile in both F_1_ hybrids and their allopolyploid derivatives have been revealed in several allopolyploid systems, while a small but significant proportion of loci display nonadditive methylation in the hybrids, and most of them are maintained in polyploids [5], [6], [14], [52], [53]. So the genome merger is the main reason for the methylation change, while genome duplication results in a secondary effect on methylation, with reversion to additivity at some loci and novel methylation status at others [6], [53]. The global patterns of DNA methylation change in *Senecio* hybrids and polyploids strongly mirror the global changes in gene expression from microarray analyses [18], indicating a possible underlying causation [53]. Even more, the nonadditivity of gene expression and DNA methylation in this system results from hybridization but can be partially reduced because of genome duplication, indicating the ameliorating effect of genome duplication on the hybridization [18]. The similar frequencies of three types of the change in three pairs of hybrids and allotetraploids (A.B/AA.BB, A.C/AA.CC, B.C/BB.CC) for total and each genome showed the main role of genome merger in genome restructuring at three levels, while further alterations at low extents occurred on genome duplication. Though some allotetraploids had lower but insignificant rates than hybrids, the ameliorating effect of genome duplication on the hybridization needs to confirm. As the variable correlations among the changes at three levels for the total and each genome, the relationships between the changes of methylation and gene expression may also vary, depending on the combinations and types of genomes. In future, it is worthwhile to make the genome-wide analysis of DNA methylation and gene expression changes in our hybrids and allopolyploids to reveal the cytoplasm and genomic effects on the reprogramming of gene expression networks and the mechanisms for their heterosis [54].

### DNA Sequence Elimination and Meiotic Diploidization in Allopolyploids

The early genetic changes in newly synthesized allopolyploids involving loss of non-coding, repetitive DNA regions from one or both parental genomes were considered to differentiate homoeologous chromosomes and to contribute to the diploidization process of allopolyploids which cannot be revealed in the natural counterparts [11], [13], [19], [55]. Recombination between parental genomes in allopolyploids is another process which may serve to further distinguish the parental genomes and also the loss of non-coding repetitive DNA in wheat allopolyploids [12], [13], [14]. Non-reciprocal translocations between parental homoeologues were found to occur at each successive generation in synthetic *B. napus* [6]. In our synthetic polyploids (AA.BB, BB.CC, AA.CC/CC.AA), degree of relationship between the parental genomes was positively correlated with the frequency of genomic changes (Table 1 and Table S2), and also with meiotic regularity [33], because AA.BB and BB.CC with more divergent genomes showed more loss of DNA bands but lower rates of homoeologous pairing than AA.CC/CC.AA with closer genomes. The higher degree of divergence was also showed by F_5_ plants from synthetic *B. juncea* compared with F_2_ plants than those from *B. napus*, resulting from genome change accumulated during the successive generations [26]. These results seem to contrast the idea above, for more sequence elimination and change were expected or needed to appear in AA.CC/CC.AA to differentiate their closely related parental genomes for the insurance of regular meiotic pairing, while less change was needed for the diploid-like pairing of divergent genomes in AA.BB and BB.CC. It was interesting that the C-genome chromosomes generally showed more normal pairing than those of A and B genomes [33], while C-genome presented the lowest rates of DNA band loss (Table 1). Probably, the sequence elimination is more extensive at initial stage of allopolyploids with more divergent genomes, such as *B. juncea*, mainly to reduce the difference of parental genomes and to coordinate the developmental process of the newly formed polyploids. However, the sequence elimination caused by hybridization and genome duplication and by meiotic recombination contributes to the cytological diploidization of the synthetic polyploids with less divergent genomes, such as *B. napus*, to avoid the homoeologous shuffling from mispairing [7], [33]. The homoeologous recombination likely played a more important role for the sequence removal in synthetic *B. napus* than *B. juncea* and *B. carinata*, because of the higher frequency of homoeologous pairing between A and C genomes than between A and B or between B and C genomes, while other non-meiosis-driven process including the activation of transposable elements might be mainly responsible for the genomic change in the latter two allotetraploids. It is perceivable that the synthetic *B. napus* takes longer time for meiotic diploidization than *B. juncea* and *B. carinata*. The further study of genetic change and meiotic behavior in our synthetic *Brassica* allopolyploids with genomes of different divergence will provide more useful information on their stabilization.

Finally, it should be pointed out that the tissue culture and subculturing and also colchicine treatment on media may induce somatic mutations which made some contributions to the genetic changes in the serial hybrids and allotetraploids observed, besides genome merger and doubling. In another aspect, the use of dominant AFLP markers is prone to misinterpretation, particularly for homoeologous locus amplification in *Brassica* species. So it is necessary to further investigate the genetic variations in these hybrids by other technology, such as NGS sequencing.

## Supporting Information

Table S1 **Genome-specific and shared bands in diploids amplified by three methodologies.**
(DOC)

Table S2 **Number and percentage of genome-specific absent and novel AFLP bands in hybrids and allotetraploids.**
(DOC)

Table S3 **Number and percentage of genome-specific absent and novel mAFLP bands in hybrids and allotetraploids.**
(DOC)

Table S4 **Number and percentage of genome-specific absent and novel cDNA-AFLP bands in hybrids and allotetraploids.**
(DOC)

Table S5 **Sequences of adaptors and primers used for pre-amplification and selective amplification in mAFLP.**
(DOC)
