# Supplementary material for: Cytoplasmic and Genomic Effects on Non-Meiosis-Driven Genetic Changes in Brassica Hybrids and Allotetraploids from Pairwise Crosses of Three Cultivated Diploids
Source: PLoS One. 2013 May 31;8(5):e65078. doi: 10.1371/journal.pone.0065078 (PMC3669095; doi:10.1371/journal.pone.0065078)
Supplement: Table S1 — Genome-specific and shared bands in diploids amplified by three methodologies. (DOC) [file pone.0065078.s001.doc]

**Table S1** Genome-specific and shared bands in diploids amplified by three methodologies

| Methods | Parental genomes | A | B | C | AB | AC | BC | ABC | Total |
| --- | --- | --- | --- | --- | --- | --- | --- | --- | --- |
| AFLP | AA | 298(48.53) | - | - | 59(9.61) | 173(28.18) | - | 84(13.68) | 614 |
|  | BB | - | 531(67.56) | - | 59(7.51) | - | 112(14.25) | 84(10.69) | 786 |
|  | CC | - | - | 461(55.54) | - | 173(20.84) | 112(13.49) | 84(10.12) | 830 |
| mAFLP | AA | 55(49.55) | - | - | 9(8.11) | 30(27.03) | - | 17(15.32) | 111 |
|  | BB | - | 74(62.71) | - | 9(7.63) | - | 18(15.25) | 17(14.41) | 118 |
|  | CC | - | - | 107(62.21) | - | 30(17.44) | 18(10.47) | 17(9.88) | 172 |
| cDNA-AFLP | AA | 117(31.20) | - | - | 28(7.47) | 105(28.00) | - | 125(33.33) | 375 |
|  | BB | - | 190(46.91) | - | 28(6.91) | - | 62(15.31) | 125(30.86) | 405 |
|  | CC | - | - | 180(38.14) | - | 105(22.25) | 62(13.14) | 125(26.48) | 472 |

A, B, C: A, B, C genome specific fragments, respectively; AB, AC, BC: fragments common to both the parental A and B, A and C, B and C genomes, respectively; ABC: fragments common to parental A, B and C genomes.
