# Supplementary material for: Cytoplasmic and Genomic Effects on Non-Meiosis-Driven Genetic Changes in Brassica Hybrids and Allotetraploids from Pairwise Crosses of Three Cultivated Diploids
Source: PLoS One. 2013 May 31;8(5):e65078. doi: 10.1371/journal.pone.0065078 (PMC3669095; doi:10.1371/journal.pone.0065078)
Supplement: Table S2 — Number and percentage of genome-specific absent and novel AFLP bands in hybrids and allotetraploids. (DOC) [file pone.0065078.s002.doc]

**Table S2** Number and percentage of genome-specific absent and novel AFLP bands in hybrids and allotetraploids

| Combinations | AFLP fragments (%) | | | | | | | | |  | | Additive fragments | | | | | | | |
| --- | --- | --- | --- | --- | --- | --- | --- | --- | --- | --- | --- | --- | --- | --- | --- | --- | --- | --- | --- |
|  | Novel | A^-^ | B^-^ | C^-^ | AB^-^ | AC^-^ | BC^-^ | ABC^-^ | Total |  |  | A | B | C | AB | AC | BC | ABC | Total |
| BB.A | 15(1.56) | 97(32.55) | 124(23.35) | - | 11 | 55 | 23 | 3  5 | 25.79 |  | | 201 | 407 | - | 48 | 118 | 89 | 81 | 1272 |
| A.B | 23(2.47) | 70(23.49) | 191(35.97) | - | 7 | 34 | 43 |  | 29.14 |  | | 228 | 340 | - | 52 | 139 | 69 | 79 | 1280 |
| AA.BB | 19(2.05) | 74(24.83) | 190(35.78) | - | 6 | 31 | 42 | 5 | 28.76 |  | | 224 | 341 | - | 53 | 142 | 70 | 79 | 1276 |
| CC.B | 8(0.70) | - | 186(35.03) | 42(9.11) | 27 | 24 | 8 | 0 | 20.66 |  | | - | 345 | 419 | 32 | 149 | 104 | 84 | 1428 |
| B.C | 6(0.51) | - | 146(27.50) | 62(13.45) | 13 | 23 | 4 | 2 | 17.95 |  | | - | 385 | 399 | 46 | 150 | 108 | 82 | 1426 |
| BB.CC | 5(0.43) | - | 161(30.32) | 67(14.53) | 15 | 21 | 4 | 3 | 19.37 |  | | - | 370 | 394 | 44 | 152 | 108 | 81 | 1425 |
| CC.A | 12(1.22) | 114(38.26) | - | 54(11.71) | 21 | 11 | 13 | 3 | 19.02 |  | | 184 | - | 407 | 38 | 162 | 99 | 81 | 1199 |
| A.C | 19(1.87) | 97(32.55) | - | 59(12.80) | 15 | 8 | 11 | 0 | 17.33 |  | | 201 | - | 402 | 44 | 165 | 101 | 84 | 1206 |
| AA.CC | 18(1.79) | 90(30.20) | - | 66(14.32) | 17 | 11 | 15 | 0 | 18.01 |  | | 208 | - | 395 | 42 | 162 | 97 | 84 | 1205 |
| CC.AA | 12(1.19) | 97(32.55) | - | 57(12.36) | 15 | 7 | 17 | 0 | 17.10 |  | | 201 | - | 404 | 44 | 166 | 95 | 84 | 1199 |
| C.A.B | 13(0.94) | 92(30.87) | 179(33.71) | 54(11.71) | 8 | 7 | 5 | 1 | 20.74 |  | | 206 | 352 | 407 | 51 | 166 | 107 | 83 | 1731 |
| A.C.B | 20(1.50) | 108(36.24) | 181(34.09) | 95(20.61) | 6 | 11 | 6 | 0 | 24.57 |  | | 190 | 350 | 366 | 53 | 162 | 106 | 84 | 1738 |
| Total | 170(1.31) | 839(31.28) | 1358(31.97) | 556(13.40) | 161(22.74) | 243(11.71) | 191(14.21) | 22(2.18) | 21.61 | | 1843 | | 2890 | 3593 | 547 | 1833 | 1153 | 986 | 16385 |

A^-^, B^-^, C^-^: A, B, C genome-specific fragments lost, respectively; AB^-^, AC^-^, BC^-^, ABC^-^: fragments lost common to two or three genomes; %novel = novel/(novel+ no change) ×100, %elimination=(specific type of parental bands eliminated)/(total of specific type of parental bands detected) ×100, %change = total change bands/total bands ×100
