# Supplementary material for: Cytoplasmic and Genomic Effects on Non-Meiosis-Driven Genetic Changes in Brassica Hybrids and Allotetraploids from Pairwise Crosses of Three Cultivated Diploids
Source: PLoS One. 2013 May 31;8(5):e65078. doi: 10.1371/journal.pone.0065078 (PMC3669095; doi:10.1371/journal.pone.0065078)
Supplement: Table S3 — Number and percentage of genome-specific absent and novel mAFLP bands in hybrids and allotetraploids. (DOC) [file pone.0065078.s003.doc]

**Table S3** Number and percentage of genome-specific absent and novel mAFLP bands in hybrids and allotetraploids

| Combinations | mAFLP fragments（%） | | | | | | | | |  | Additive Methylation fragments | | | | | | | |
| --- | --- | --- | --- | --- | --- | --- | --- | --- | --- | --- | --- | --- | --- | --- | --- | --- | --- | --- |
|  | Novel | A- | B- | C- | AB- | AC- | BC- | ABC- | Total |  | A | B | C | AB | AC | BC | ABC | Total |
| BB.A | 6(3.66) | 17(30.91) | 12(16.22) | - | 1 | 8 | 3 | 4  1 | 24.40  19.42 |  | 38 | 62 |  | 8 | 22 | 15 | 13 | 209 |
| A.B | 3(1.78) | 14(25.45) | 16(21.62) | - | 1 | 3 | 2 |  |  |  | 41 | 58 |  | 8 | 27 | 16 | 16 | 206 |
| AA.BB | 5(2.86) | 10(18.18) | 11(14.86) | - | 1 | 8 | 2 | 1 | 18.27 |  | 45 | 63 |  | 8 | 22 | 16 | 16 | 208 |
| CC.B | 2(0.91) | - | 15(20.27) | 12(11.21) | 1 | 5 | 1 | 4 | 15.56 |  |  | 59 | 95 | 8 | 25 | 17 | 13 | 257 |
| B.C | 2(0.87) | - | 12(16.22) | 13(12.15) | 1 | 2 | 0 | 0 | 11.67 |  |  | 62 | 94 | 8 | 28 | 18 | 17 | 257 |
| BB.CC | 2(0.54) | - | 9(12.16) | 12(11.21) | 0 | 1 | 0 | 2 | 10.12 |  |  | 65 | 95 | 9 | 29 | 18 | 15 | 257 |
| CC.A | 1(1.92) | 24(43.64) | - | 17(15.89) | 1 | 5 | 1 | 3 | 21.94 |  | 31 |  | 90 | 8 | 25 | 17 | 14 | 237 |
| A.C | 1(0.50) | 17(30.91) | - | 12(11.21) | 3 | 1 | 3 | 0 | 15.61 |  | 38 |  | 95 | 6 | 29 | 15 | 17 | 237 |
| AA.CC | 1(0.51) | 18(32.73) | - | 12(11.21) | 3 | 0 | 6 | 2 | 17.72 |  | 37 |  | 95 | 6 | 30 | 12 | 15 | 237 |
| CC.AA | 0(0) | 23(41.82) | - | 15(14.02) | 1 | 3 | 3 | 1 | 19.49 |  | 32 |  | 92 | 8 | 27 | 15 | 16 | 236 |
| C.A.B | 1(0.4) | 18(32.73) | 19(25.68) | 21(19.63) | 0 | 3 | 0 | 0 | 19.94 |  | 37 | 55 | 86 | 9 | 27 | 18 | 17 | 324 |
| A.C.B | 4(1.56) | 16(29.09) | 19(25.68) | 20(18.69) | 0 | 1 | 0 | 1 | 19.43 |  | 39 | 55 | 87 | 9 | 29 | 18 | 16 | 330 |
| Total | 28(1.13) | 157(31.72) | 113(19.09) | 134(13.91) | 13(12.04) | 40(11.11) | 21(9.72) | 19(9.31) | 17.71 |  | 338 | 479 | 829 | 95 | 320 | 195 | 185 | 2966 |

A^-^, B^-^, C^-^: A, B, C genome-specific mAFLP fragments lost, respectively; AB^-^, AC^-^, BC, ABC^--^: methylation fragments lost common to two or three genomes; %novel = novel/(novel+ no change) ×100, %elimination=(specific type of parental bands eliminated)/(total of specific type of parental bands detected) ×100, %change =total change bands/total bands ×100
