# Supplementary material for: Cytoplasmic and Genomic Effects on Non-Meiosis-Driven Genetic Changes in Brassica Hybrids and Allotetraploids from Pairwise Crosses of Three Cultivated Diploids
Source: PLoS One. 2013 May 31;8(5):e65078. doi: 10.1371/journal.pone.0065078 (PMC3669095; doi:10.1371/journal.pone.0065078)
Supplement: Table S4 — Number and percentage of genome-specific absent and novel cDNA-AFLP bands in hybrids and allotetraploids. (DOC) [file pone.0065078.s004.doc]

**Table S4** Number and percentage of genome-specific absent and novel cDNA-AFLP bands in hybrids and allotetraploids

| Combinations | cDNA-AFLP fragments(%) | | | | | | | | |  | Additive fragments | | | | | | | |
| --- | --- | --- | --- | --- | --- | --- | --- | --- | --- | --- | --- | --- | --- | --- | --- | --- | --- | --- |
|  | Novel | A^-^ | B^-^ | C^-^ | AB^-^ | AC^-^ | BC^-^ | ABC^-^ | Total |  | A | B | C | AB | AC | BC | ABC | Total |
| BB.A | 3(0.51) | 16(13.68) | 13(6.84) | - | 1 | 14 | 1 | 0 | 7.62 |  | 101 | 177 |  | 27 | 91 | 61 | 125 | 630 |
| A.B | 4(0.70) | 15(12.82) | 28(14.74) | - | 1 | 13 | 1 | 1 | 9.98 |  | 102 | 162 |  | 27 | 92 | 61 | 124 | 631 |
| AA.BB | 3(0.51) | 8(6.84) | 30(15.79) | - | 0 | 7 | 1 | 1 | 7.94 |  | 109 | 160 |  | 28 | 98 | 61 | 124 | 630 |
| CC.B | 2(0.32) | - | 32(16.84) | 14(7.78) | 7 | 8 | 11 | 4 | 11.27 |  | - | 158 | 166 | 21 | 97 | 51 | 121 | 692 |
| B.C | 1(0.16) | - | 20(10.53) | 19(10.56) | 2 | 14 | 1 | 1 | 8.39 |  | - | 170 | 161 | 26 | 91 | 61 | 124 | 691 |
| BB.CC | 1(0.16) | - | 29(15.26) | 15(8.33) | 1 | 12 | 1 | 0 | 8.54 |  | - | 161 | 165 | 27 | 93 | 61 | 125 | 691 |
| CC.A | 0(0) | 20(17.09) | - | 8(4.44) | 2 | 0 | 0 | 0 | 4.86 |  | 97 |  | 172 | 26 | 105 | 62 | 125 | 617 |
| A.C | 0(0) | 15(12.82) | - | 13(7.22) | 3 | 3 | 11 | 3 | 7.78 |  | 102 |  | 167 | 25 | 102 | 51 | 122 | 617 |
| AA.CC | 0(0) | 21(17.95) | - | 18(10.00) | 5 | 2 | 2 | 3 | 8.26 |  | 96 |  | 162 | 23 | 103 | 60 | 122 | 617 |
| CC.AA | 1(0.18) | 11(9.40) | - | 11(6.11) | 6 | 7 | 15 | 7 | 9.39 |  | 106 |  | 169 | 22 | 98 | 47 | 118 | 618 |
| C.A.B | 2(0.28) | 9(7.69) | 54(28.42) | 19(10.56) | 3 | 6 | 10 | 3 | 13.10 |  | 108 | 136 | 161 | 25 | 99 | 52 | 122 | 809 |
| A.C.B | 2(0.28) | 21(17.95) | 42(22.11) | 19(10.56) | 3 | 4 | 8 | 4 | 12.73 |  | 96 | 148 | 161 | 25 | 101 | 54 | 121 | 809 |
| Total | 19(0.26) | 136(12.92) | 248(16.63) | 136(8.40) | 34(10.12) | 90(7.14) | 62(8.33) | 27(1.80) | 9.34 |  | 917 | 1272 | 1484 | 302 | 1170 | 682 | 1473 | 8052 |

A^-^, B^-^, C^-^: A, B, C genome-specific cDNA-AFLP fragments lost, respectively; AB^-^, AC^-^, BC^-^, ABC: cDNA-AFLP fragments lost common to two or three C genomes; %novel = novel/(novel+ no change) ×100, %elimination=(specific type of parental bands eliminated)/(total of specific type of parental bands detected) ×100, %change =total change bands/total bands ×100.
