# Supplementary material for: Cytoplasmic and Genomic Effects on Non-Meiosis-Driven Genetic Changes in Brassica Hybrids and Allotetraploids from Pairwise Crosses of Three Cultivated Diploids
Source: PLoS One. 2013 May 31;8(5):e65078. doi: 10.1371/journal.pone.0065078 (PMC3669095; doi:10.1371/journal.pone.0065078)
Supplement: Table S5 — Sequences of adaptors and primers used for pre-amplification and selective amplification in mAFLP. (DOC) [file pone.0065078.s005.doc]

**Table S5** Sequences of adaptors and primers used for pre-amplification and selective amplification in mAFLP

| Adaptors/Primers | Abbreviations | Sequence(5’*-*3’) |
| --- | --- | --- |
| *EcoR*I-adapter1 | Ead1  Ead2 | CTC GTA GAC TGC GTA CC |
| *EcoR*I-adapter2 |  | AAT TGG TAC GCA GTC TAC |
| *Pst*I-adapter1 | Pad1 | CTC GTA GAC TGC GTA CAT GCA |
| *Pst*I -adapter2 | Pad2 | TGT ACG CAG TCT AC |
| *Pst*I -adapter*1 | Pad*1 | GCA TCA GTG CAT GCG TGC A |
| *Pst*I -adapter*2 | Pad*2 | CGC ATG CAC TGA TG |
| *Mse*I-adapter1 | Mad1 | GAC GAT GAG TCC TGA G |
| *Mse*I-adapter2 | Mad2 | TAC TCA GGA CTC AT |
| *Mse*I-adapter+1 | Mad+1 | CTC GTA GAC TGC GTA CC |
| *Mse*I-adapter+2 | Mad+2 | T AG GTA CCG AGT C |
| *Mse*I-Pre-selective primers | M00 | GAT GAG TCC TGA GTA AC |
| *Mse*I-Pre-selective primers+ | M00+ | GTA GAC TGC GTA CCT AA |
| *Pst*I -pre-selective primers | P00 | GAC TGC GTA CAT GCA G |
| *Mse*I-pre-selective primers-AA | M01 | GAT GAG TCC TGA GTA ACAA |
| *Mse*I-pre-selective primers-TC | M02 | GAT GAG TCC TGA GTA ACTC |
| *Mse*I-pre-selective primers-AT | M03 | GAT GAG TCC TGA GTA ACAT |
| *Mse*I-pre-selective primers-GG | M04 | GAT GAG TCC TGA GTA ACGG |
| *Mse*I-pre-selective primers-AC | M05 | GAT GAG TCC TGA GTA ACAC |
| *Mse*I-pre-selective primers-TG | M06 | GAT GAG TCC TGA GTA ACTG |
| *Mse*I-pre-selective primers-GT | M07 | GAT GAG TCC TGA GTA ACGT |
| *Mse*I-pre-selective primers-AG | M08 | GAT GAG TCC TGA GTA ACAG |
| *Mse*I-pre-selective primers-TA | M09 | GAT GAG TCC TGA GTA ACTA |
| *Mse*I-pre-selective primers-GA | M10 | GAT GAG TCC TGA GTA ACGA |
| *Pst*I -pre-selective primers-CG | P01 | GAC TGC GTA CAT GCA GCG |
| *Pst*I -pre-selective primers-GT | P02 | GAC TGC GTA CAT GCA GGT |
| *Pst*I-pre-selective primers-TC | P03 | GACTGC GTA CAT GCA GTC |
| *Pst*I-pre-selective primers-AAT | P04 | GAC TGC GTA CAT GCA GAAT |
| *Pst*I-pre-selective primers-GGA | P05 | GAC TGC GTA CAT GCA GGGA |
| *Pst*I-pre-selective primers-AAT | P06 | GAC TGC GTA CAT GCA GATT |
| *Pst*I-pre-selective primers-ACG | P07 | GAC TGC GTA CAT GCA GACG |
| *Pst*I-pre-selective primers-TAC | P08 | GAC TGC GTA CAT GCA GTAC |
| *Pst*I-pre-selective primers-CGA | P09 | GAC TGC GTA CAT GCA GCGA |
| *Pst*I-pre-selective primers-TCG | P10 | GAC TGC GTA CAT GCA GTCG |
